# Paediatric Ketamine Bladder Case Report: A Burgeoning Problem in the Paediatric Population

**DOI:** 10.1155/crpe/2254453

**Published:** 2026-08-31

**Authors:** Shreya Kulkarni, Arjun Visa, Marita Isaac, Joanne Parr, Charlotte Dunford E., Ravindar Anbarasan, Azad Mathur, Milind Kulkarni

**Affiliations:** ^1^ Department of Paediatric Urology, Jenny Lind Hospital, Norfolk and Norwich University Hospital, Norwich, England, UK, nhs.uk; ^2^ Department of Plastics Surgery, Leicester Royal Infirmary, University Hospitals of Leicester, Leicester, England, UK, nhs.uk; ^3^ Department of Urology, Norfolk and Norwich University Hospital, Norwich, England, UK, nhs.uk; ^4^ Norwich Medical School, University of East Anglia, Norwich, England, UK, uea.ac.uk

**Keywords:** case report, ketamine abuse, ketamine-induced uropathy, paediatrics

## Abstract

Ketamine misuse is an increasing public health concern among adolescents in the United Kingdom, with initiation reported as early as 14 years of age and a rapidly rising demand for drug and alcohol support services. Chronic ketamine misuse is associated with significant urological sequelae, including ketamine‐induced uropathy, a condition increasingly encountered within paediatric and adolescent populations. Despite this trend, there remains a lack of unified paediatric‐specific management guidelines. We present the case of a 15‐year‐old boy who was referred urgently with abdominal pain and haematuria. Imaging revealed a thick‐walled, irregular bladder with vascular soft tissue plaques, alongside a significantly raised albumin–creatinine ratio. Further assessment identified a history of heavy recreational ketamine use (up to 2 g/day) commencing at age 14. Following engagement with community drug and alcohol support services, the patient significantly reduced ketamine use, with subsequent improvement in symptoms, highlighting the potential reversibility of disease with early intervention and abstinence. This case underscores the emerging burden of ketamine‐induced uropathy in paediatric urology and the need for a structured, age‐appropriate management approach. Drawing on existing adult consensus guidance, we propose a tailored paediatric framework incorporating early noninvasive investigations, stepwise escalation of treatment, multidisciplinary involvement and close collaboration with youth‐focused substance misuse services. Increased reporting and research into paediatric ketamine uropathy are essential to clarify disease trajectory, inform clinical guidelines and support public health strategies aimed at reducing long‐term urological morbidity in young people.

## 1. Introduction

Ketamine misuse has been a growing problem amongst adult and young adult populations. Globally, countries have seen a rise in the use of ketamine in nightlife and youth culture [1].

In the United Kingdom, ketamine abuse poses a significant concern in the adolescent population, with age of initiation typically as young as 14 [2]. An anonymised study by NHS England over 185 schools found 11% of 15‐year‐olds had been offered ketamine at some point [3]. The number of young people seeking drug and alcohol services for ketamine doubled from 512 in 2022 to 1465 in 2025 [4], making it the third most common problem substance to lead to treatment.

Ketamine misuse allows users to undergo detachment and a feeling of euphoria. It is easily available at a relatively low cost, and its more relaxed restrictions as a Class C drug until 2013 have contributed to its lack of stigma in comparison to other illegal substances.

Due to the growing nature of ketamine use in the paediatric population (less than 16 years old), clinical departments are now seeing a rise in the sequelae associated with the misuse. Chronic misuse can lead to ketamine‐induced uropathy, with symptoms that may include acute urinary retention, bladder cramps, pelvic pain, dysuria and hematuria [5, 6]. Cystoscopy findings include thickening and irregularity of the bladder wall and ulcerations.

Paediatric urology departments in the United Kingdom have started to establish specialised clinics to tackle the growing problem of ketamine‐induced uropathy [7].

We describe here the presentation and management of ketamine‐induced uropathy in an under 16‐year‐old patient secondary to substance misuse.

## 2. Case Presentation

**FIGURE 1:**
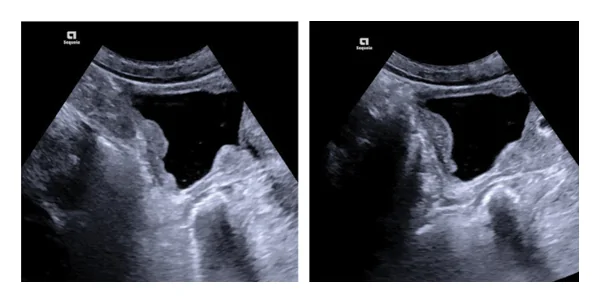
Ultrasound scan of the bladder at the time of diagnosis. Ultrasound showing thickened and irregular bladder walls and small capacity.

A 15‐year‐old boy was referred urgently to the paediatric surgical team with a history of epigastric abdominal pain, radiating to the right lumbar region with associated visible haematuria. He was investigated with ultrasound and was found to have a thick‐walled, irregular bladder, with plaques of vascular soft tissue within (Figure 1), consistent with ketamine‐induced uropathy.

On re‐review, he was noted to have no further abdominal pain, though ongoing nonvisible haematuria. He was also found to have a high albumin–creatinine ratio (ACR) at 79.4 mg/mmol. He was noted to use recreational drugs, though initially denied ongoing use.

As we suspected there could be ketamine/drug use implications, we brought him to our joint transition clinic, which involved paediatric and adult urologists, paediatric and adult specialist nurses and a transition nurse. For his appointment, a specialist substance abuse nurse and a community psychiatrist with a specialist interest in substance abuse were also invited. The boy was jointly evaluated.

On further in‐depth review of the history, he had been using ketamine, up to 2 g per day, from the age of 14. However, after turning 15, the patient found support with local charities and was able to cut down use dramatically. Besides urgency, most of the other symptoms had subsided. Communication with the community support worker was initiated, along with advice regarding good urological care, regular contact with the substance misuse nurse and contact with the charitable Matthew project. A routine ultrasound KUB was organised.

Impact of ketamine on bladder and kidneys was discussed in detail with him. He was appropriately counselled by the psychologist and urologists together.

**FIGURE 2:**
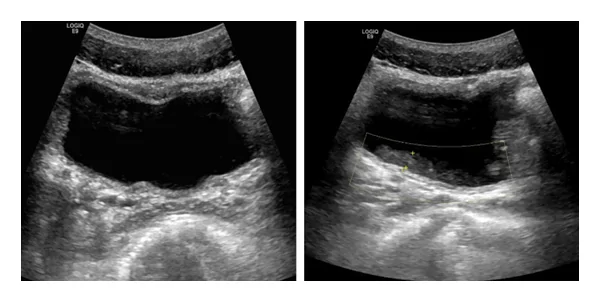
Ultrasound of the bladder after cessation period, demonstrating improvement.

After complete cessation of ketamine, he stated that his bladder was a lot better and he had improved weight gain. He was able to hold urine for up to 3 hours a day, and during nighttime, he needed to get up once, depending on how much he had to drink. He did not have any other symptoms. Repeat ultrasound demonstrated improved appearance of the bladder with a few plaque‐like thickenings of the bladder wall. He continues to have microscopic haematuria, but renal function is preserved (Figure 2).

He remains under surveillance with the adult urology team in the ketamine clinic and has ongoing psychology support in the community.

## 3. Discussion

Currently, there are well‐laid‐out guidelines for the management of ketamine uropathy in adults, with a stepwise approach [8]. There is no unified paediatric urology management guideline, despite the rising number of young adults and children at risk of ketamine misuse. Literature on cases and management of ketamine uropathy are few in paediatrics and limited to medical uses of ketamine [9, 10]. Literature on paediatric presentations associated with misuse of the drug is now emerging [11].

In conjunction with the BJUI Consensus 2024, we propose the need for a management guideline when considering paediatric patients presenting with ketamine‐associated uropathy.

Those who require urological services should have a tailored paediatric approach. A high index of suspicion is required with increasing awareness of the symptoms related to ketamine bladder from GPs, paediatricians/paediatric nephrologists and paediatric surgeons. We suggest that paediatric patients specifically should be evaluated in a multidisciplinary clinic (such as transitional clinics) with the input of paediatric and adult urologists, paediatric and adult specialist nurses and psychiatrists with a specialist interest in de‐addiction services. Further input and follow‐up from community paediatricians and paediatric and adult nephrology may be required. This multispoke approach factors in family and social influence strongly.

History from the patient should carefully assess the duration of drug abuse and the dosage regularly taken. Younger patients may be less forthcoming about their ketamine use, especially in the presence of parents or guardians. Any suspected cases of ketamine induced uropathy should have toxicology screening for ketamine levels, which often requires specific screening assays on request.

Urological symptoms can be assessed through specific questionnaires (such as ICIQ –LUTS International Consultation on Incontinence Questionnaire–Lower Urinary Tract Symptoms).

Urine analysis with culture, cytology and baseline bloods (FBCs, U&Es and LFTs) should be performed in all cases [8].

We recommend that initial investigations should include baseline USS imaging and noninvasive bladder assessment (including uroflowmetry, postmicturition ultrasound scan and bladder diary).

Key initial management steps include cessation of ketamine misuse, symptomatic management with analgesics and appropriate psychological support for de‐addiction.

Treatment options should escalate as symptoms advise, from medical management, invasive testing if persistent (including MCUG and or/urodynamic investigations) and surgical management, with invasive management reserved for the most severe as per the Adult BAUS guidelines [8].

We feel there is an importance in direct connection with paediatric/young people–focused drug and alcohol services and charities to aid recovery in a specialised, paediatric fashion. As in the case of our young patients, these can aid in supporting the cessation of drug misuse and can also support young adults when they face transition into adult care. Accessing community‐based and charitable sources can be a barrier for young patients to receive the appropriate care they require, and streamlining this as part of their healthcare may improve their engagement.

Further research and documentation of potential cases of paediatric ketamine bladder will help to determine the nature of this disease. Whether there is relative plasticity to recovery from ketamine abuse, as suggested by the recovery during a period of abstinence in our patient, or increased risk of significant need for surgical intervention (such as increased need for bladder/ureteric or kidney‐based interventions such as bladder injection, bladder augmentation, ureteric stenting, or kidney diversion including but not limited to nephrostomy use), is yet to be established.

More focus on cases of paediatric ketamine–induced uropathy may aid the production of public health messaging and policy to reduce a potentially large burden of urological disease onto a young adult population.

## Funding

No funding was received for this study.

## Consent

Written informed consent for publication of clinical details and/or clinical images was obtained from the patient and the patient’s parent or legal guardian.

## Conflicts of Interest

The authors declare no conflicts of interest.

## Supporting Information

Additional supporting information can be found online in the Supporting Information section.

## Supporting information


**Supporting Information** CARE‐checklist‐English‐2013.

## Data Availability

The data that support the findings of this study are available on request from the corresponding author. The data are not publicly available due to privacy or ethical restrictions.
