# Supplementary material for: Paediatric Ketamine Bladder Case Report: A Burgeoning Problem in the Paediatric Population
Source: Case Rep Pediatr. 2026 Aug 31;2026:2254453. doi: 10.1155/crpe/2254453 (PMC13530406; doi:10.1155/crpe/2254453)
Supplement: Supplementary file 1 [file CRPE-2026-2254453-s001.pdf]

CARE case report guidelines

CARE Checklist of information to include when writing a case report

(cc) BY-NC-ND

Topic	Item	Checklist item description	Reported on Line
Title	1	The diagnosis or intervention of primary focus followed by the words "case report"	Line 1
Key Words	2	2 to 5 key words that identify diagnoses or interventions in this case report, including "case report"	Line 7 - 11
Abstract (no references)	3a	Introduction: What is unique about this case and what does it add to the scientific literature?	Line 18
	3b	Main symptoms and/or important clinical findings	Line 20 - 22
	3c	The main diagnoses, therapeutic interventions, and outcomes	Line 22 - 26
	3d	Conclusion—What is the main "take-away" lesson(s) from this case?	Line 27 - 34
Introduction	4	One or two paragraphs summarizing why this case is unique (**may include references**)	Line 46 - 56
Patient Information	5a	De-identified patient specific information.	Line 59
	5b	Primary concerns and symptoms of the patient.	Line 59 - 60
	5c	Medical, family, and psycho-social history including relevant genetic information	Line 66
	5d	Relevant past interventions with outcomes	N/A
Clinical Findings	6	Describe significant physical examination (PE) and important clinical findings.	Line 64 - 66
Timeline	7	Historical and current information from this episode of care organized as a timeline	Line 73 - 86
Diagnostic Assessment	8a	Diagnostic testing (such as PE, laboratory testing, imaging, surveys).	Line 61, 65
	8b	Diagnostic challenges (such as access to testing, financial, or cultural)	Line 66
	8c	Diagnosis (including other diagnoses considered)	Line 62
	8d	Prognosis (such as staging in oncology) where applicable	N/A
Therapeutic Intervention	9a	Types of therapeutic intervention (such as pharmacologic, surgical, preventive, self-care)	Line 77 - 80
	9b	Administration of therapeutic intervention (such as dosage, strength, duration)	N/A
	9c	Changes in therapeutic intervention (with rationale)	N/A
Follow-up and Outcomes	10a	Clinician and patient-assessed outcomes (if available)	Line 83
	10b	Important follow-up diagnostic and other test results	Line 88
	10c	Intervention adherence and tolerability (How was this assessed?)	Line 83
	10d	Adverse and unanticipated events	N/A
Discussion	11a	A scientific discussion of the strengths AND limitations associated with this case report	Line 95 - 141
	11b	Discussion of the relevant medical literature **with references**.	Line 95 - 141
	11c	The scientific rationale for any conclusions (including assessment of possible causes)	N/A
	11d	The primary "take-away" lessons of this case report (without references) in a one paragraph conclusion	Line 150
Patient Perspective	12	The patient should share their perspective in one to two paragraphs on the treatment(s) they received	N/A
Informed Consent	13	Did the patient give informed consent? Please provide if requested	Yes ☒ No ☐
